# Genetic strategy to decrease complement activation with adenoviral therapies

**DOI:** 10.1371/journal.pone.0215226

**Published:** 2019-04-26

**Authors:** Christopher M. Gentile, Anton V. Borovjagin, Jillian R. Richter, Aditi H. Jani, Hongju Wu, Kurt R. Zinn, Jason M. Warram

**Affiliations:** 1 Department of Otolaryngology, University of Alabama at Birmingham, Birmingham, Alabama, United States of America; 2 Department of Biomedical Engineering, University of Alabama at Birmingham, Birmingham, Alabama, United States of America; 3 Department of Surgery, University of Alabama at Birmingham, Birmingham, Alabama, United States of America; 4 University School of Medicine at University of Alabama at Birmingham, Birmingham, Alabama, United States of America; 5 Department of Medicine, Tulane University, Tulane, Louisiana, United States of America; 6 Department of Radiology, Michigan State University, East Lansing, Michigan, United States of America; Sechenov First Medical University, RUSSIAN FEDERATION

## Abstract

**Background:**

A major obstacle to using recombinant adenoviral vectors in gene therapy is the natural ability of human adenovirus to activate the classical and alternate complement pathways. These innate immune responses contribute to hepatic adenoviral uptake following systemic delivery and enhance the humoral immune responses associated with adenoviral infection.

**Methods:**

A recombinant Ad5 vector was genetically modified to display a peptide sequence (“rH17d’”), a known inhibitor of the classical complement pathway. The replication-defective vectors Ad5.HVR2-rH17d’ and Ad5.HVR5-rH17d’ were constructed by engineering the rH17d’ peptide into the hypervariable region (HVR)-2 or HVR5 of their major capsid protein hexon. Control Ad5 vectors were created by incorporation of a 6-histidine (His_6_)-insert in either HVR2 or HVR5 (Ad5.HVR2-His_6_ and Ad5.HVR5-His_6_, respectively). All vectors encoded CMV promoter-controlled firefly luciferase (Luc). The four vectors were evaluated in TIB76 mouse liver cells and immunocompetent mice to compare infectivity and liver sequestration, respectively.

**Results:**

*In vitro* studies demonstrated that preincubation of all the Ad5 vectors with fresh serum significantly increased their gene transfer relative to preincubation with PBS except Ad5.HVR5-rH17d’, whose infectivity of liver cells showed no serum-mediated enhancement. In line with that, mice injected with Ad5.HVR2-rH17d’ or Ad5.HVR5-rH17d’ showed significantly lower luciferase expression levels in the liver as compared to the respective control vectors, whereas efficiency of tumor transduction by rH17d’ and His_6_ vectors following their intratumoral injection was similar.

**Conclusions:**

Displaying a complement-inhibiting peptide on the Ad5 capsid surface by genetic modification of the hexon protein could be a suitable strategy for reducing Ad5 liver tropism (Ad5 sequestration by liver), which may be applicable to other gene therapy vectors with natural liver tropism.

## Introduction

Activation of innate immunity and induction of inflammation are major obstacles for the use of adenoviral vectors for human gene therapy.[1] Systemic intravascular delivery of human adenovirus type 5(Ad5) rapidly induces a dose-dependent toxic response that is transcription-independent and mediated by activation of the complement system,[2,3] production of proinflammatory cytokines/chemokines,[4] and blood-based coagulation factors.[5] These innate mechanisms lead not only to potentially deadly host responses such as liver damage and coagulopathy, but also to sequestration of gene delivery vectors from circulation, preventing their transduction of target tissues. Modification of the Ad hexon to evade the natural innate immune response to Ad5 is a promising strategy for improving the safety and clinical utility of Ad5-based gene therapies.[6]

Efforts in improving gene delivery by using modified Ad vectors have led to intense study of the mechanisms that govern the Ad-associated innate immune response. In this regard, Ad type 5 (Ad5) vectors, in particular, have been extensively studied given their broad utility in gene therapy applications. Conventionally, following intravascular administration, Ad5 vectors immediately come into contact with a variety of blood cells, plasma proteins and coagulation factors that mediate and regulate viral tropism and biodistribution in vivo. In humans as well as animal models, most of the systemically administered Ad5 virus is sequestered by the liver, and therefore, Ad transgene expression is localized predominantly in hepatocytes.[7] The complement system plays a significant role in the inherent Ad5 liver tropism in mice, as demonstrated by decreased liver transduction in complement component 3 (C3)-deficient animals, as compared to wild type mice following intravenous Ad5 delivery.[2] Cichon *et al*. also showed that complement was activated in a majority of human plasma samples when challenged with different adenoviral serotypes, indicating a likely role of complement in dictating the human response to Ad as well.[8]

Given that the complement pathway is a regulator of Ad5 liver tropism, it was reasoned that a strategy to modify the Ad5 hexon to reduce complement response might also reduce Ad5 liver sequestration and transgene expression. To this end, the hexon of a replication-defective Ad5 vector was modified with the recombinant peptide rH17d’, a known and potent inhibitor of the classical complement pathway.[9] Modifications were made at either hypervariable region-2 (HVR2) or HVR5 since these domains are recognized as suitable loci for optimal surface exposure of inserted epitopes.[10,11]

The recombinant peptide rH17d is a potent complement inhibitor produced by duplicating the C-terminal motifs (^17^H-^26^Y) of Sh-CRIT-ed1, a fragment of the membrane protein Sh-CRIT, present on the plasma membrane of the *Schistosoma* parasite. Both rH17d and Sh-CRIT-ed1 exhibit sequence homology with the beta-chain of C4 (C4b). In the complement cascade, C4b binds to C2, which is subsequently cleaved to form the active C3 convertase complex, C4b2a. Following cleavage by C4b2a, C3b binds to the microbial surface tagging the pathogen for phagocytosis. The homology of rH17d with C4b makes this recombinant peptide a strong inhibitor of the classical complement pathway by interfering with the formation of the C4b2a complex and preventing subsequent formation of the C3b fragment required for opsonization and pathogen removal.

In the present work, the immune response to rH17d-modified Ad5 vectors was evaluated by *in vitro* and *in vivo* analyses. A modified version of rH17d (herein called rH17d’) was constructed using a 36-amino acid protein sequence (LGS-HEVKIKHFSPY-HEVKIKHFSPY-GS-HHHHHH-LGS) that was inserted into either the HVR2 or HVR5 of the hexon protein, resulting in the vectors Ad5.HVR2-rH17d’ and Ad5.HVR5-rH17d’, respectively. Control Ad5 vectors were constructed by insertion of a His_6_ sequence (LGS-HHHHHH-LGS) at either HVR2 or HVR5 yielding Ad5.HVR2-His_6_ or Ad5.HVR5-His_6_, respectively. All experimental and control Ad5 vectors contained a CMV-controlled firefly luciferase (Luc) reporter, which was used to track vector transduction by using bioluminescence imaging (BLI).

## Materials and methods

### Construction of adenoviral vectors

Ad5.HVR2-His_6_ and Ad5.HVR5-His_6_ vectors were constructed as describes previously.[10] In order to incorporate the rH17d’ complement inhibitory peptide into the HVR2 and HVR5 of the Ad5 hexon protein, the oligos encoding the rH17d’ amino acid sequence were inserted into the parental hexon shuttle vectors HVR2-His_6_/pH5S and HVR5-His_6_/pH5S.[10] Specifically, rH17d’ sense oligo (5' Phos-GA TCC CAC GAA GTG AAA ATT AAA CAC TTT TCT CCG TAT CAC GAA GTG AAA ATT AAA CAC TTT TCT CCG TAT G 3') and anti-sense oligo (5' Phos-GA TCC ATA CGG AGA AAA GTG TTT AAT TTT CAC TTC GTG ATA CGG AGA AAA GTG TTT AAT TTT CAC TTC GTG G 3') were annealed and ligated into partially BamHI-digested HVR2-His_6_/pH5S and HVR5-His_6_/pH5S, resulting in the formation of HVR2-rH17d’/pH5S and HVR5-rH17d’/pH5S, respectively. Of note, there were two BamHI sites in the parental shuttle vectors, therefore, partial digestion was required to obtain linearized vectors at the desired BamHI site within the corresponding HVR-His_6_ regions. The positive clones were identified by restriction digestion and further confirmed by sequencing analysis. Next, the shuttle vectors HVR2-rH17d’/pH5S and HVR5-rH17d’/pH5S were recombined with the Ad5 backbone plasmid pAd5/DH (GL) via homologous recombination in bacterial BJ5183 cell strain.[10,12] After the positive clones carrying rH17d’-modified Ad5 genome, namely pAd5.HVR2-rH17d’ (GL) and pAd5.HVR5-rH17d’ (GL), were identified, they were subjected to the virus rescue procedure. Because the E1 region in the backbone pAd5/DH (GL) was replaced with CMV-promoter-driven GFP and firefly Luc genes (as reporters), the resulting viral vectors were replication-deficient. To rescue the Ad5.HVR2-rH17d’ (GL) and Ad5.HVR5-rH17d’ (GL) viruses, the modified Ad5 genomes in the corresponding rescue vectors were released by PacI digestion, purified and transfected into the helper HEK293 cells stably expressing Ad5-E1 proteins. Upon formation of the viral plaques the primary viral lysates were harvested, freeze-thawed 3 times and used for re-infection of a large number of HEK293 cells in order to propagate and purify the recombinant viruses by CsCl gradient centrifugation as described previously.[12]

### SDS-PAGE analysis of viral proteins

Equal numbers of CsCl-purified viral particles (10^10^ VP) of each viral preparation were mixed with Laemmli sample buffer and denatured by boiling for 5 minutes. The samples were then separated on a 4–15% gradient polyacrylamide SDS gels (SDS-PAGE) and stained with Gelcode® Blue Stain Reagent (ThermoScientific, Rockford, IL) according to the manufacturer’s protocol.

### In vitro experiments

Mouse liver hepatoma (TIB76) cells were incubated with rH17d’ and His_6_ Ad vectors following a 5-minute pre-incubation with serum collected from C57BL/6 mice. For each Ad5 vector, the infectivity level was first determined in PBS and used as baseline for normalization of infectivity levels in the presence of serum. In brief, 5 x10^8^ VP were mixed with 100 μL of serum (or PBS) at 37°C followed by dilution in serum-free MEM. Diluted Ad vectors (5 x10^7^ VP) were then added to confluent TIB76 cells in 24-well plates and incubated for 1 hour at 37°C with gentle rocking. Following incubation, Ad vector solutions were removed and the cells washed with PBS. Normal media was added to the cells, and infectivity was determined 24 hours post-treatment by quantifying intracellular expression of the firefly Luc transgene by using the IVIS-100 CCD imaging system (Caliper Life Sciences, Mountain View, CA). Prior to bioluminescence imaging, 0.1 mg/mL luciferin in normal media was added to the cells. Matched region of interest (ROI) analysis was performed using instrument software (Living Image 3.2) to quantify total luciferase counts per well.

### ELISAs

Adenoviral vectors (2.6 x 10^8^ VP) were diluted in sterile PBS and immobilized on Nunc Maxisorp Immunoplates II microtiter plates (Fisher Scientific, Atlanta, GA) by 5-hour incubation at room temperature. After extensive washes with 0.05% Tween-20 in PBS (PBS-T), wells were blocked for 1 hour with 1% BSA in Borate saline (BS-BSA; pH 8.4). Serum from Ad-injected mice was collected at 14 days post-immunization, pooled group-wise, serially-diluted in BS-BSA, added to the Ad-coated plates, and incubated overnight at 4°C. After washing with PBS-T, alkaline phosphatase (AP)-conjugated goat anti-mouse IgG or IgM antibodies (1:2000 dilution in PBS-T; Southern Biotechnology Associates, Birmingham, AL) were added for 4 hours at room temperature. Unbound antibodies were removed by extensive washing with PBST, followed by a 20-minute incubation with the AP substrate p-Nitrophenylphosphate (1 mg/mL in PBST, Sigma). The absorbance at 405 nm (OD405) was measured using a plate reader (Molecular Devices, Sunnyvale, CA).

### Animal experiments

Animal studies were performed in accordance with the National Institutes of Health recommendations and the approval of the Institutional Animal Care and Use Committee at the University of Alabama at Birmingham. For immunization, C57BL/6J mice (male, 7–10 weeks, Jackson Laboratory, Bar Harbor, ME) were injected intravenously with 4 x10^9^ VP of Ad5.HVR2-rH17d’, Ad5.HVR5-rH17d’, Ad5.HVR2-His_6_ or Ad5.HVR5-His_6_. Mice were sequentially imaged with BLI for a 30-day time period using the IVIS-100 CCD imaging system following systemic anesthesia with isofluorane by inhalation and intraperitoneal injection of 2.5 mg luciferin. Matched ROI analysis was performed to quantify liver luciferase expression. Separate mice were similarly immunized with the Ad5 vectors for serum collection at 14 days. For *in vitro* studies and for baseline IgG/IgM measurements, serum was collected from Ad-naive mice.

A-427 lung carcinoma epithelial cells (American Type Culture Collection, Manassas, VA) were used to grow flank tumors in athymic male nude mice (7–10 weeks, Frederick Cancer Research, Hartford, CT). Mature tumors were injected intra-tumorally (IT) with 1.25 x 10^9^ VP of Ad5.HVR2-rH17d’, Ad5.HVR5-rH17d’, Ad5.HVR2-His_6_ or Ad5.HVR5-His_6_. Luciferase expression in the tumors was monitored using BLI for 10 days and quantified using matched ROI analyses.

### Statistical analysis

Results are reported as the mean plus or minus standard error of the mean (SEM). Data were analyzed for statistical significance by Student’s t-test or analysis of variance (ANOVA) with Bonferroni’s multiple comparison test, using Prism (version 6.0, GraphPad Software) where appropriate.

## Results

**Fig 1:**
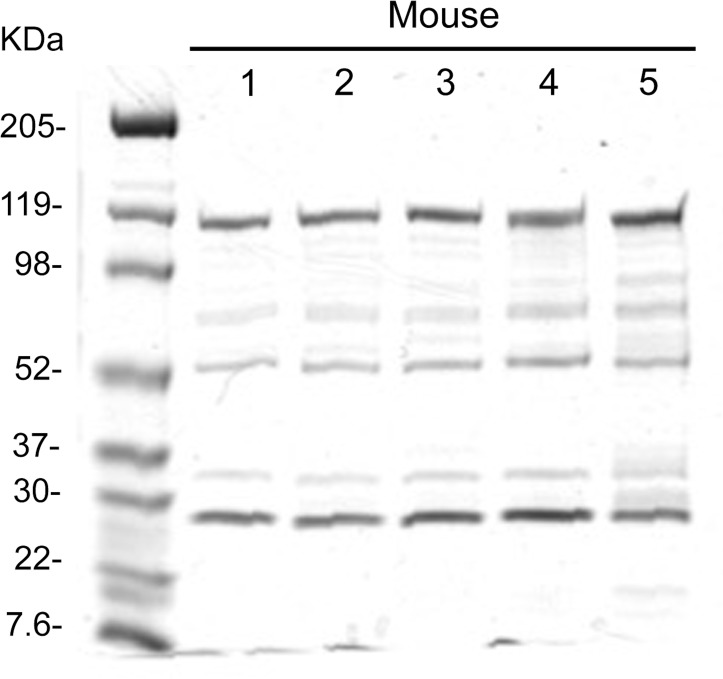
SDS-PAGE analysis of the rH17d-containing Ad5 vectors. In the assay, 10^10^ VP of CsCl-purified viral vectors were subjected to SDS-PAGE analysis. Gelcode staining of the viral capsid proteins showed that viral particles of the rH17d’-containing Ad5 vectors had correct protein composition, as compared to the control (unmodified and His_6_-modified) vectors. The relative intensities of the protein bands in each lane (virus sample) were consistent with viral titration data (physical titer values). M: molecular weight marker; Lanes: 1—Ad5 (GL); 2—Ad5.HVR2-His_6_ (GL); 3—Ad5.HVR2-rH17d’ (GL); 4—Ad5.HVR5-His_6_ (GL); 5—Ad5.HVR5-rH17d’ (GL).

SDS-PAGE analysis (**Fig 1**) shows characteristic banding pattern for adenoviral proteins. The top band (~119 kDa) represents the hexon protein, whose size is consistent between all modified and unmodified Ad vectors since neither the rH17d’ or His_6_ epitope insertion causes a visible change in the hexon protein size. Gelcode gel staining also demonstrates consistency between gel-loaded amounts of all the viruses and their physical titers, estimated at different times, since loading of the same number of viral particles (VP; 10^10^ VP) of each viral preparation resulted in similar intensities of the major protein bands on the gel.

**Fig 2:**
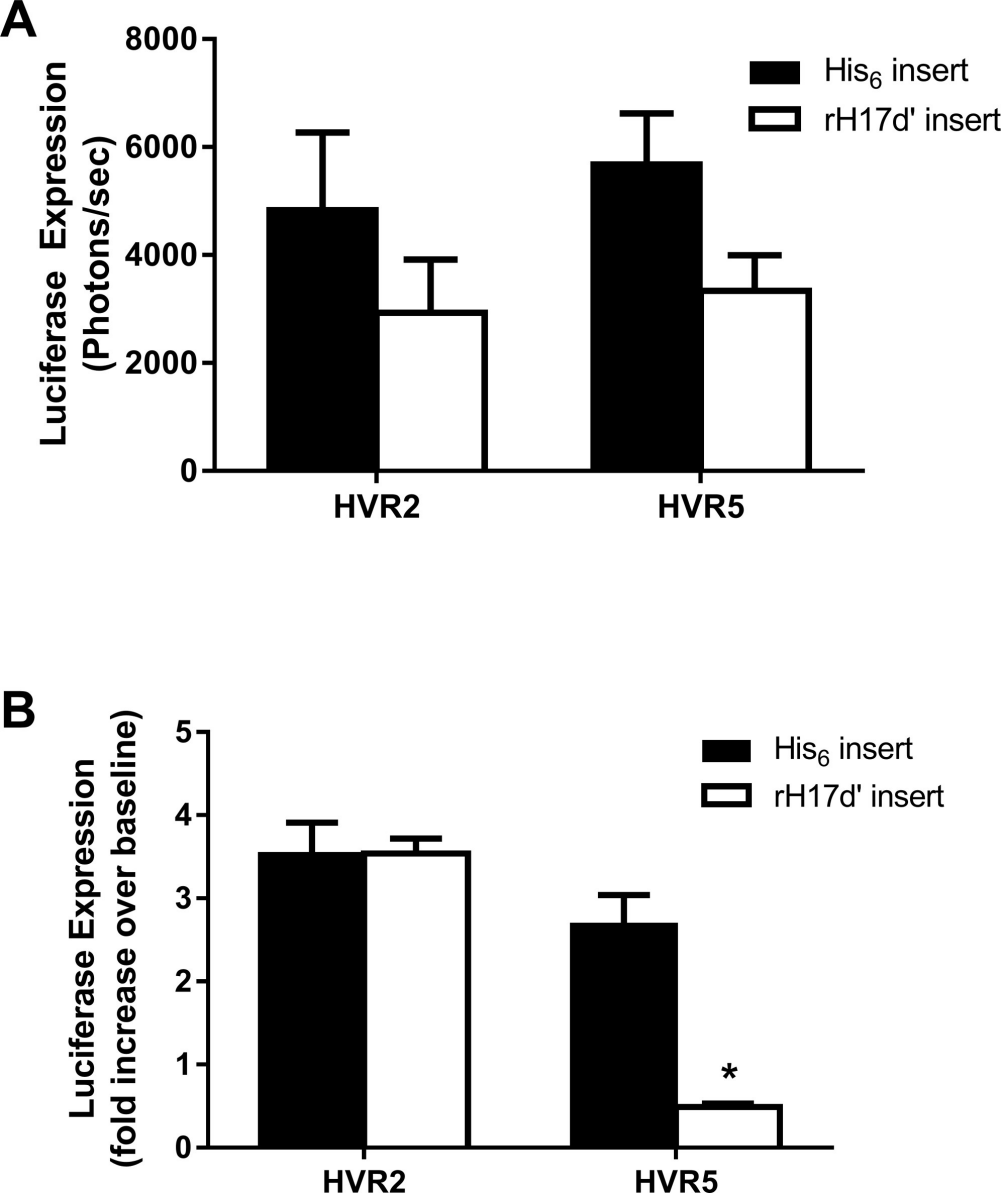
Liver cell infectivity of rH17d-containing Ad5 vectors. (A) Baseline infectivity of the Ad5 vectors was established by incubating TIB76 liver cells with rH17d’- or His_6_-modified adenoviruses for 1 h in PBS (5 x10^8^ VP). Infectivity levels were determined by quantification of luciferase transgene expression in infected cells 24h post-transduction. (B) The Ad5 vectors were also pre-incubated with serum from normal C57BL/6 mice and then used to infect TIB76 liver cells. Luciferase transgene expression in virus-infected cells was quantified and normalized to the respective infectivity levels observed in PBS (without pre-incubation with serum). All data represent the mean ± SEM from 3 independent experiments and 4 replicates (n = 4) each; *p<0.001 relative to HVR5-His_6_ control.

Baseline infectivity levels for the rH17d’- and His_6_-modified Ad5 vectors were estimated by measuring luciferase transgene expression in liver cells following their incubation with each virus in PBS (**Fig 2A**). Although no statistically significant differences in the baseline infectivity levels were observed between the HVR2- or HVR5-modified vectors, insertion of rH17d’ at either HVR2 or HVR5 locales significantly reduced transgene expression compared with the respective controls. To test the effect of factors naturally present in blood on infectivity of rH17d’ Ad5 vectors in cultured liver cells, the Ad5 vectors were preincubated with serum collected from normal C57BL/6 mice prior to cell transduction. As shown in **Fig 2B**, liver cells transduced with the Ad5 vectors containing either His_6_ or rH17d’ at HVR2 (Ad5.HVR2-His_6_ or Ad5.HVR2-rH17d’) exhibited a 3.5-fold increase in luciferase expression over baseline levels (in PBS) following incubation with serum, using an equivalent multiplicity of infection for both Ad vectors. On the other hand, vector with rH17d’ at HVR5 (Ad5.HVR5-rH17d’) demonstrated a significantly decreased (~5-fold) expression of the luciferase transgene as compared to the HVR5 His_6_-control vector (Ad5.HVR5-His_6_). Given the known role of rH17d’in inhibiting the classical complement cascade (Oh, 2003), these data indicate that rH17d’ incorporated into the Ad5 hexon at HVR5, but not HVR2, could possibly reduce complement-mediated transduction of TIB76 liver cells *in vitro*, although other mechanisms could be involved as well.

**Fig 3:**
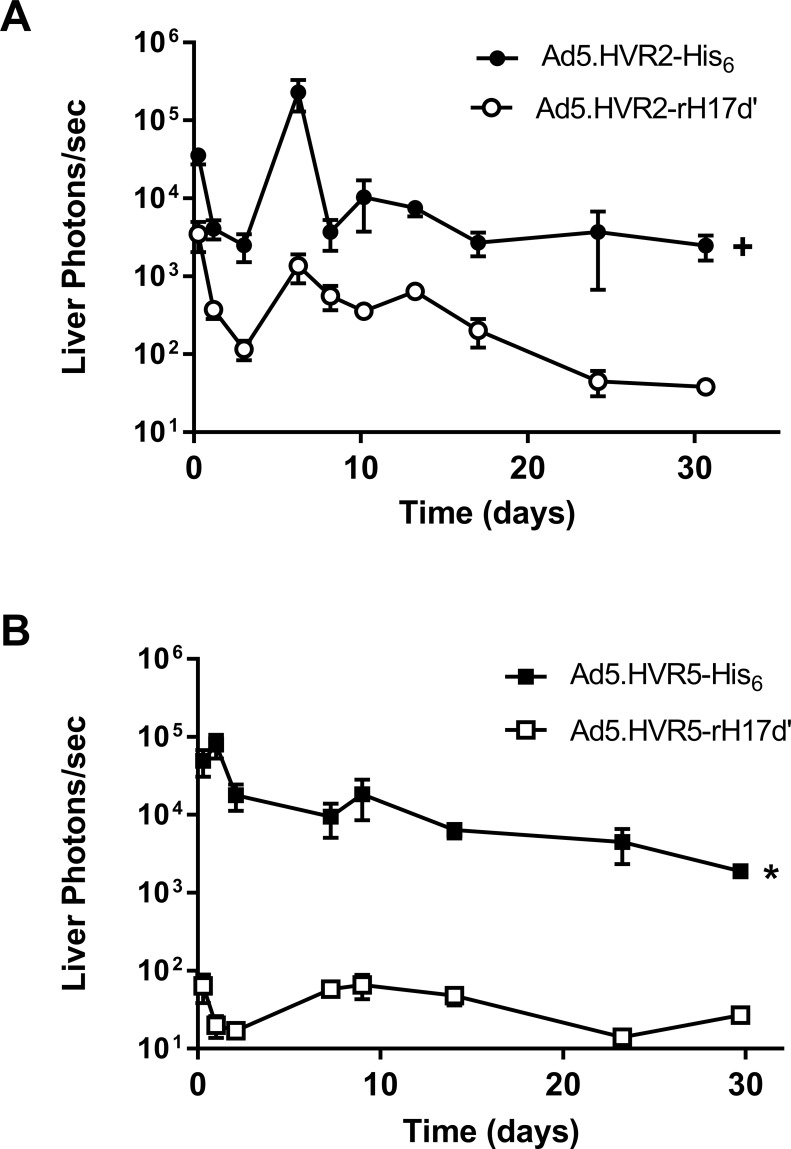
Luciferase expression in mice after intravenous injection rH17d’-Ad5 vectors as revealed by live BLI. 4 x 10^9^ VP of (A) Ad5.HVR2-rH17d’ and Ad5.HVR2-His_6_ or (B) Ad5.HVR5-rH17d’ and Ad5.HVR5-His_6_ were intravenously administered via tail vein injection and live BLI was repeatedly performed for 30 days to quantify luciferase expression in mouse livers. All data represent the mean ± SEM (n = 5); +p<0.01 relative to Ad5.HVR2-rH17d’ for all time points and *p<0.001 relative to Ad5.HVR5-rH17d’ for all time points.

*In vivo* expression of luciferase reporter in the liver following intravenous injection of mice with Ad5.HVR2-rH17d’ or Ad5.HVR5-rH17d’ vectors, shown in **Fig 3,** was significantly reduced, as compared with the respective His_6_-control vectors. However, incorporation of rH17d’ in HVR5 resulted generally in a much greater reduction in Ad5 liver transduction as compared to that of the Ad5 vectors with rH17d’ in HVR2. While the reduction in liver transduction level for Ad5.HVR2-rH17d’ ranged from 6-fold (on day 8) to 83-fold (on day 24), for Ad5.HVR5-rH17d’ this decrease ranged from 70-fold (at day 27) to 3900-fold (on day 1).

**Fig 4:**
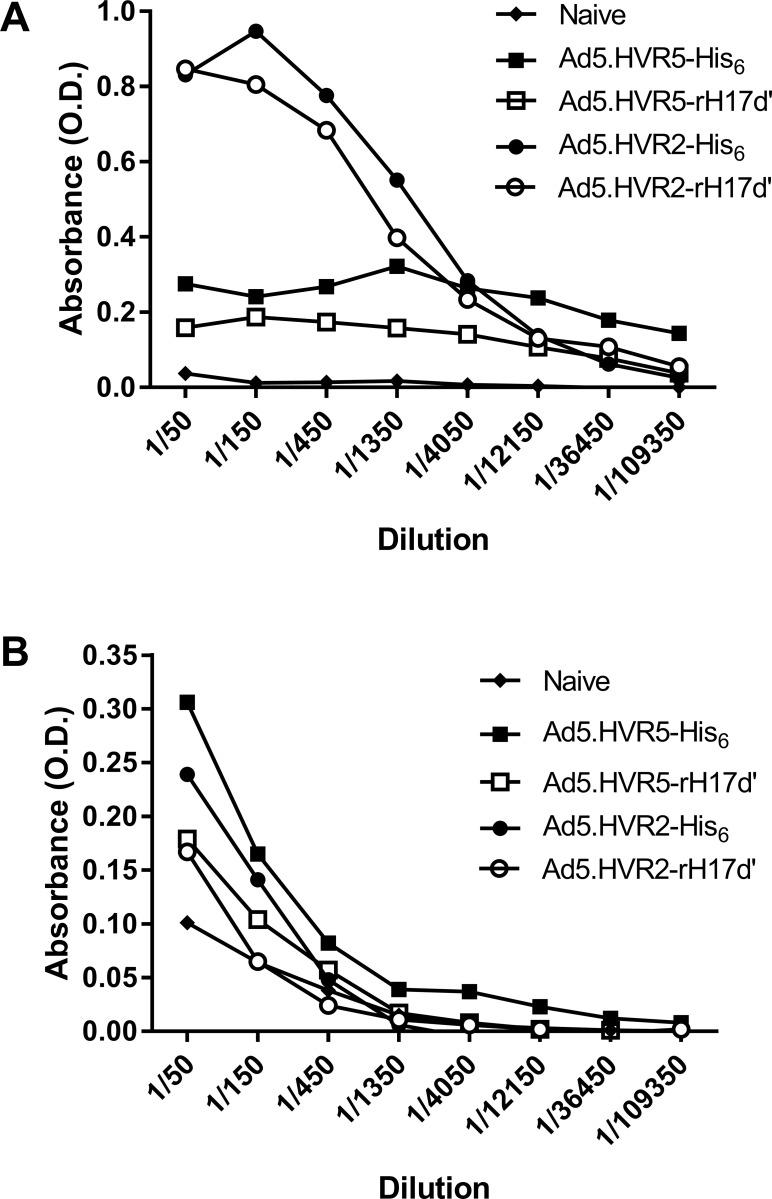
Humoral immune response to Ad5 vectors carrying rH17d’ or His_6_ peptide modifications on their capsid surfaces. Mice were immunized with 4 x 10^9^ VP of each adenoviral vectors, followed by serum collection 14 days later for quantification of (A) IgG and (B) IgM levels. Control (base line) values for IgG and IgM were obtained from naïve (non-immunized) mice.

To explore the humoral immune response to the rH17d’-Ad5 vectors, immunocompetent mice were immunized by intravascular injection of the rH17d’- or the His_6_-modified Ad5 vectors. Fourteen days after immunization, serum IgG levels (Ad-specific antibody titers) were found substantially lower in mice immunized with the HVR5-modified Ad5 vectors as compared to the IgG levels measured in mice, exposed to the HVR2-modified vectors. Although for both HVR5 and HVR2 hexon modification locales, the rH17d’ insertion somewhat reduced IgG levels relative to those observed for the His_6_-insertion in the respective hexon modification sites (**Fig 4A**), the magnitude of the difference was moderate and its biological meaningfulness and statistical significance remained unclear. In contrast, the HVR5-modified vectors were significantly less immunogenic and induced a substantially (4 to 5-fold) lower IgG levels, than both vectors carrying insertions in HVR2 region of the capsid protein hexon. This suggests a more important role of the hexon’s HVR5 locale-associated epitope, as compared to that of HVR2, for the Ad5 *in vivo* immunogenicity. For all groups, IgM levels on day 14 post-immunization were only slightly reduced relative to 6His control levels (**Fig 4B**). Our results thus indicate that the IgG level reduction effect is rather related to HVR5 epitope alteration (distortion) per se, than to insertion of a specific amino acid sequence (rH17d’ or His_6_) into that locale.

**Fig 5:**
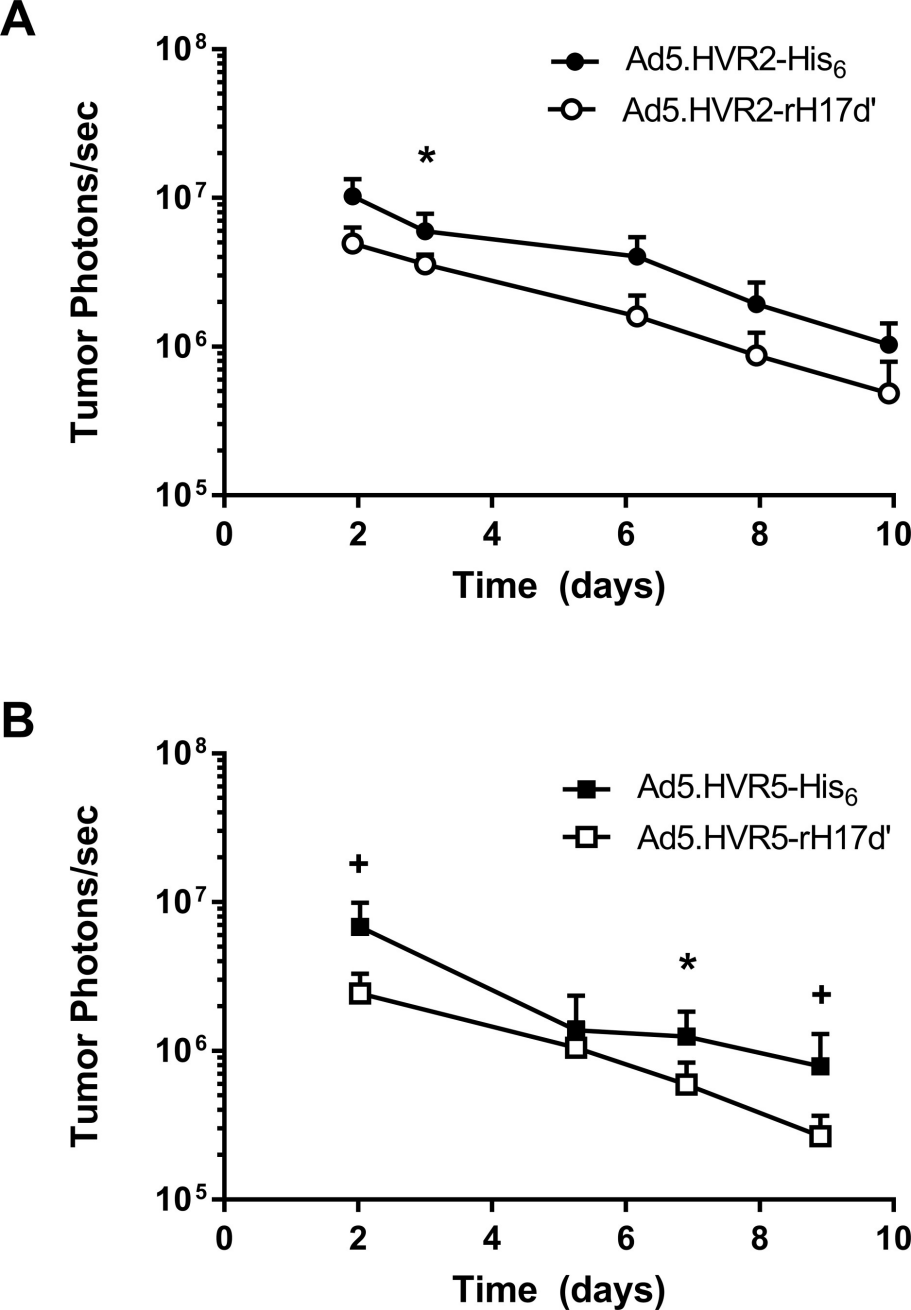
Luciferase reporter expression analysis in A427-derived xenograft tumors following IT injection of rH17d’-Ad5 vectors. 2.5 x 10^9^ VP of (**A**) Ad5.HVR2-rH17d’ and Ad5.HVR2-His_6_ and (**B**) Ad5.HVR5-rH17d’ and Ad5.HVR5-His_6_ were injected directly into xenograft tumors established in nude mice. All data represent the mean ± SEM (n = 7); *p<0.05 and +p<0.01 relative to respective rH17d’ vectors at the corresponding time point.

The infectivity levels of the rH17d’-Ad5 vectors for A-427 lung carcinoma xenograft tumors established in nude mice were evaluated by intratumoral (IT) injection of the Ad5 vectors followed by BLI of Luc transgene expression. **Fig 5** shows that both vectors containing the rH17d’ modification of the hexon (Ad5.HVR2-rH17d’ and Ad5.HVR5-rH17d’) exhibit *in viv*o transduction levels slightly below their respective His_6_-Ad5 controls. As expected for replication incompetent adenoviruses, luciferase expression levels readily declined over the 10 day evaluation period as tumor cells continued to proliferate resulting in a reduction of transgene expression.

## Discussion

Activation of the complement pathway is a potentially adverse response to adenovirus-based gene therapy. Strategies to down-regulate the complement pathway could minimize the undesired consequences that compromise the safety and efficacy of gene delivery vectors. In this study, a genetically-modified adenovirus that displayed on its capsid surface numerous copies of a peptide, known to inhibit the classical complement pathway, was evaluated for its ability to reduce unwanted liver transduction.

Rux *et al*. suggested that various serotypes of Ad could be grouped according to sequence variations in the structural protein hexon’s hypervariable regions (HVRs), named from HVR1 to HVR7.[13] Furthermore, in line with those phylogenetic variations, the HVR2 and HVR5 were found to tolerate incorporation of foreign peptides and identified as hexon locales optimal for antigen display on the surface of the Ad5 capsid,[10,11,14,15,16,17,18,19] providing rationale for the design of the adenoviral vectors evaluated in this study. Therefore, it was reasoned that insertion of a peptide in these hexon locales to down-regulate complement would be both structurally and functionally tolerated by the Ad5 vector. Another advantage of using hexon for the peptide display is the large number of these molecules making up the Ad capsid (240 hexon trimers), the total number of inserts that could be displayed in a single viral particle is 240 ×3 = 720 copies.

In the present work, the peptide display on the Ad capsid was achieved by genetic modification of the Ad5 hexon DNA coding sequence to generate hexon chimeras that were incorporated into the viral capsid during replication and packaging of the Ad5 vector. The foreign peptide used for insertion in HVR2 or HVR5 was a modified version of rH17d (named rH17d’), which itself is a homodimer of the 11 C-terminal amino acids of Sh-CRIT-ed1[9] with sequence similarity to the beta-chain of human and mouse C4b. This sequence is known to down-regulate complement by interfering with the formation of the C3 convertase C4b2a. Characterization of the capsid-modified recombinant Ad5 vectors using SDS-PAGE demonstrated that neither the rH17d’ insert nor the control His_6_ insert altered the size of the hexon protein, thus, all the vectors evaluated in this work contained similarly sized structural proteins (**Fig 1**). A CMV-controlled firefly luciferase transgene was incorporated in the recombinant Ad5 genomes to easily monitor gene delivery as a result of vector transduction.

Incorporation of rH17d’ into the Ad5 vectors was shown to decrease liver cell transduction both *in vitro* and *in vivo* (**Figs 2B and 3**). *In vitro* experiments suggested that components naturally present in serum augment liver cell transduction above the baseline (PBS) levels, whereas insertion of rH17d’ at HVR5 significantly inhibits this effect. *In vivo*, liver transduction by both Ad5.HVR2-rH17d’ and Ad5.HVR5-rH17d’ was significantly reduced as well, although the relative decrease in luciferase expression in liver tissue was overall greater for Ad5 vectors with HVR5-inserted rH17d’ peptide.

Quantification of Ad5-specific antibodies in mice pre-immunized with rH17d’-Ad5 vectors demonstrated lower levels of IgG production (**Fig 4**), suggesting that the rH17d’ peptide exposed on the Ad5 capsid surface not only lowers the transduction efficiency of liver cells *in vitro* and *in vivo*, but also reduces the acquired immune response to systemically administered Ad5. Given the role of complement as a functional bridge between the innate and adaptive immune responses,[20] it is not surprising that inhibition of complement activation by rH17d’ could also suppresses Ad-specific antibody responses, resulting in decreased IgG levels in plasma.

Although the mechanisms responsible for the reduced liver tropism of the rH17d’-Ad5 vectors were not fully elucidated in this study, the *in vitro* data suggest that rH17d’ modification of the hexon itself reduces Ad5 infectivity for TIB76 liver cells (**Fig 2A**) in addition to possible inhibition of complement-mediated mechanisms of liver transduction when the modification is placed at HVR5 (**Fig 2B**). The greater reduction in liver tropism observed for Ad5.HVR5-rH17d’, as compared to Ad5.HVR2-rH17d’ (**Fig 3**), may therefore be explained by an additive effect of two independent mechanisms: one involving hexon modification itself and the other involving complement inhibition by rH17d’ inserted in the HVR5 locale.

Previous studies aimed at deciphering the role of complement in the mechanism of Ad liver tropism resulted in findings similar to the ones of the present study. In 2004, Zinn *et al*. used BLI to demonstrate a dose-dependent reduction in liver transduction in C3 knockout mice, as compared to wild-type mice, following intravascular injection of an Ad5-luc vector.[2] In addition to highlighting the role of complement in liver sequestration of Ad5, the above referenced study also used a fiber-fibritin Ad5chimera to demonstrate that the effect of complement on liver transduction is independent of native Ad5 receptor (Coxsackie-and-Adenovirus Receptor (CAR))-mediated mechanisms.[2] More recently, others have provided evidence that the inherent liver tropism of most Ad5 vectors used for gene therapy is due to the efficient transduction of hepatocytes, which is predominantly mediated by Ad5 binding to heparan sulfate proteoglycans (HSPGs) on liver cells via Ad5 hexon-coagulation factor X (FX) bridging interaction.[21,22,23] In relation to the findings of this study, it is plausible to suggest that in addition to inhibiting complement activation, rH17d’ modification may also interfere with FX binding to Ad5, which has been shown to involve hexon HVR loops,[18,24] thereby reducing vector transduction of hepatocytes. Alternatively, a yet unknown mechanism of liver transduction, involving a complement-binding cellular receptor, may exist.

The results of this study led to yet another important conclusion regarding the mechanism of Ad5-specific immune response. Analysis of vectors’ capability to induce production of Ad5-specific antibodies by IgG level measurement in mouse sera indicates that hexon epitope(s) of the native HVR5-loop sequence/structure, play(s) an essential role in determining Ad5 immunogenicity, as opposed to the epitope(s) of HVR2 in the Ad5 capsid.

The other important finding of this study is that the rH17d’ peptide incorporation in either of the two (HVR2 or HVR5) hexon locales did not substantially affect the ability of Ad5 to transduce tumors *in vivo*, relative to the 6His control modification, following intratumoral injection of the modified vectors (**Fig 5**). Furthermore, the experiments in A547 cells also demonstrate that the insertion of rH17d’ in HVR5 does not significantly attenuate viral infectivity in general (in a non-specific way), whereas liver cell transduction is dramatically decreased, suggesting that Ad5 transduction of lung carcinoma (A547) and liver (TIB76) cells utilizes different mechanisms. The dramatic decrease in liver transduction of Ad5.HVR5-rH17d’ *in vivo* and *in vitro* could possibly be explained by a mechanism involving complement inhibition by rH17d’ peptide inserted at HVR5. On the other hand, the tumor transduction level of Ad5.HVR2-rH17d’ was reduced less than 3-fold at day 2 relative to the His_6_ control vector. These findings are particularly important for gene therapy applications of Ad5.HVR5-rH17d’, suggesting its attenuated liver tropism and the resulting capability of partially avoiding liver sequestration, while retaining infectivity for tumor tissues. This could have significant implications for the use of Ad5.HVR5-rH17d’ as a gene therapy vector. However, further work is needed to ultimately evaluate transgene delivery to target tissues following systemic administration of the Ad5.HVR5-rH17d’ vector. Given the previously established capability of the rH17d’ peptide to inhibit complement activation [9], on the one hand, and exposure of the HVR5 epitope(s) on the Ad5 capsid surface, on the other [16], the data obtained in this study support utilization of capsid-modified Ad5 vectors with the rH17d’ peptide incorporated into the hexon’s HVR5 locale as a means to reduce Ad5-associated immune response, which may involve complement inactivation as a part of the underlying mechanism. In addition, such capsid modification strategy could also be used to reduce liver tropism of Ad5-based vectors. All the above features of Ad5.HVR5-rH17d’ may have important clinical implications for human gene therapy.

## References

[1] Hartman ZC, Appledorn DM, Serra D, Glass O, Mendelson TB, et al (2008) Replication-attenuated Human Adenoviral Type 4 vectors elicit capsid dependent enhanced innate immune responses that are partially dependent upon interactions with the complement system. Virology 374: 453–467. 10.1016/j.virol.2008.01.017 18280530 PMC2720025

[2] Zinn KR, Szalai AJ, Stargel A, Krasnykh V, Chaudhuri TR (2004) Bioluminescence imaging reveals a significant role for complement in liver transduction following intravenous delivery of adenovirus. Gene Ther 11: 1482–1486. 10.1038/sj.gt.3302331 15295616

[3] Tian J, Xu Z, Smith JS, Hofherr SE, Barry MA, et al (2009) Adenovirus activates complement by distinctly different mechanisms in vitro and in vivo: indirect complement activation by virions in vivo. J Virol 83: 5648–5658. 10.1128/JVI.00082-09 19321608 PMC2681959

[4] Zhang Y, Chirmule N, Gao GP, Qian R, Croyle M, et al (2001) Acute cytokine response to systemic adenoviral vectors in mice is mediated by dendritic cells and macrophages. Mol Ther 3: 697–707. 10.1006/mthe.2001.0329 11356075

[5] Doronin K, Flatt JW, Di Paolo NC, Khare R, Kalyuzhniy O, et al (2012) Coagulation factor X activates innate immunity to human species C adenovirus. Science 338: 795–798. 10.1126/science.1226625 23019612 PMC4762479

[6] Arnberg N (2009) Adenovirus receptors: implications for tropism, treatment and targeting. Rev Med Virol 19: 165–178. 10.1002/rmv.612 19367611

[7] Shayakhmetov DM, Gaggar A, Ni S, Li ZY, Lieber A (2005) Adenovirus binding to blood factors results in liver infection and hepatotoxicity. J Virol 79: 7478–7491. 10.1128/JVI.79.12.7478-7491.2005 15919903 PMC1143681

[8] Cichon G, Boeckh-Herwig S, Schmidt HH, Wehnes E, Muller T, et al (2001) Complement activation by recombinant adenoviruses. Gene Ther 8: 1794–1800. 10.1038/sj.gt.3301611 11803399 PMC7091591

[9] Oh KS, Kweon MH, Rhee KH, Ho Lee K, Sung HC (2003) Inhibition of complement activation by recombinant Sh-CRIT-ed1 analogues. Immunology 110: 73–79. 10.1046/j.1365-2567.2003.01706.x 12941143 PMC1783027

[10] Wu H, Han T, Belousova N, Krasnykh V, Kashentseva E, et al (2005) Identification of sites in adenovirus hexon for foreign peptide incorporation. J Virol 79: 3382–3390. 10.1128/JVI.79.6.3382-3390.2005 15731232 PMC1075677

[11] McConnell MJ, Danthinne X, Imperiale MJ (2006) Characterization of a permissive epitope insertion site in adenovirus hexon. J Virol 80: 5361–5370. 10.1128/JVI.00256-06 16699016 PMC1472126

[12] Wu H, Dmitriev I, Kashentseva E, Seki T, Wang M, et al (2002) Construction and characterization of adenovirus serotype 5 packaged by serotype 3 hexon. J Virol 76: 12775–12782. 10.1128/JVI.76.24.12775-12782.2002 12438602 PMC136697

[13] Rux JJ, Kuser PR, Burnett RM (2003) Structural and phylogenetic analysis of adenovirus hexons by use of high-resolution x-ray crystallographic, molecular modeling, and sequence-based methods. J Virol 77: 9553–9566. 10.1128/JVI.77.17.9553-9566.2003 12915569 PMC187380

[14] Vigne E, Mahfouz I, Dedieu JF, Brie A, Perricaudet M, et al (1999) RGD inclusion in the hexon monomer provides adenovirus type 5-based vectors with a fiber knob-independent pathway for infection. J Virol 73: 5156–5161. 10233980 10.1128/jvi.73.6.5156-5161.1999 PMC112562

[15] Roberts DM, Nanda A, Havenga MJ, Abbink P, Lynch DM, et al (2006) Hexon-chimaeric adenovirus serotype 5 vectors circumvent pre-existing anti-vector immunity. Nature 441: 239–243. 10.1038/nature04721 16625206

[16] Worgall S, Krause A, Qiu J, Joh J, Hackett NR, et al (2007) Protective immunity to pseudomonas aeruginosa induced with a capsid-modified adenovirus expressing P. aeruginosa OprF. J Virol 81: 13801–13808. 10.1128/JVI.01246-07 17942539 PMC2168865

[17] Matthews QL, Yang P, Wu Q, Belousova N, Rivera AA, et al (2008) Optimization of capsid-incorporated antigens for a novel adenovirus vaccine approach. Virol J 5: 98 10.1186/1743-422X-5-98 18718011 PMC2535600

[18] Waddington SN, McVey JH, Bhella D, Parker AL, Barker K, et al (2008) Adenovirus serotype 5 hexon mediates liver gene transfer. Cell 132: 397–409. 10.1016/j.cell.2008.01.016 18267072

[19] Matthews QL, Fatima A, Tang Y, Perry BA, Tsuruta Y, et al (2010) HIV antigen incorporation within adenovirus hexon hypervariable 2 for a novel HIV vaccine approach. PLoS One 5: e11815 10.1371/journal.pone.0011815 20676400 PMC2910733

[20] Dunkelberger JR, Song WC (2010) Complement and its role in innate and adaptive immune responses. Cell Res 20: 34–50. 10.1038/cr.2009.139 20010915

[21] Parker AL, Waddington SN, Nicol CG, Shayakhmetov DM, Buckley SM, et al (2006) Multiple vitamin K-dependent coagulation zymogens promote adenovirus-mediated gene delivery to hepatocytes. Blood 108: 2554–2561. 10.1182/blood-2006-04-008532 16788098

[22] Bradshaw AC, Parker AL, Duffy MR, Coughlan L, van Rooijen N, et al (2010) Requirements for receptor engagement during infection by adenovirus complexed with blood coagulation factor X. PLoS Pathog 6: e1001142 10.1371/journal.ppat.1001142 20949078 PMC2951380

[23] Xu Z, Qiu Q, Tian J, Smith JS, Conenello GM, et al (2013) Coagulation factor X shields adenovirus type 5 from attack by natural antibodies and complement. Nat Med 19: 452–457. 10.1038/nm.3107 23524342

[24] Ma J, Duffy MR, Deng L, Dakin RS, Uil T, et al (2015) Manipulating adenovirus hexon hypervariable loops dictates immune neutralisation and coagulation factor X-dependent cell interaction in vitro and in vivo. PLoS Pathog 11: e1004673 10.1371/journal.ppat.1004673 25658827 PMC4450073

